# Cu_x_Co_1-x_Fe_2_O_4_ (x = 0.33, 0.67, 1) Spinel Ferrite Nanoparticles Based Thermoplastic Polyurethane Nanocomposites with Reduced Graphene Oxide for Highly Efficient Electromagnetic Interference Shielding

**DOI:** 10.3390/ijms23052610

**Published:** 2022-02-26

**Authors:** Anju, Raghvendra Singh Yadav, Petra Pötschke, Jürgen Pionteck, Beate Krause, Ivo Kuřitka, Jarmila Vilčáková, David Škoda, Pavel Urbánek, Michal Machovský, Milan Masař, Michal Urbánek

**Affiliations:** 1 Centre of Polymer Systems, University Institute, Tomas Bata University in Zlín, Trida Tomase Bati 5678, 760 01 Zlín, Czech Republic; deswal@utb.cz (A.); kuritka@utb.cz (I.K.); vilcakova@utb.cz (J.V.); dskoda@utb.cz (D.Š.); urbanek@utb.cz (P.U.); machovsky@utb.cz (M.M.); masar@utb.cz (M.M.); murbanek@utb.cz (M.U.); 2 Leibniz Institute of Polymer Research Dresden (IPF Dresden), 01069 Dresden, Germany; poe@ipfdd.de (P.P.); pionteck@ipfdd.de (J.P.); krause-beate@ipfdd.de (B.K.)

**Keywords:** electromagnetic interference shielding, magnetic nanoparticles, reduced graphene oxide, nanocomposites, spinel ferrite

## Abstract

Cu_x_Co_1-x_Fe_2_O_4_ (x = 0.33, 0.67, 1)-reduced graphene oxide (rGO)-thermoplastic polyurethane (TPU) nanocomposites exhibiting highly efficient electromagnetic interference (EMI) shielding were prepared by a melt-mixing approach using a microcompounder. Spinel ferrite Cu_0.33_Co_0.67_Fe_2_O_4_ (CuCoF1), Cu_0.67_Co_0.33_Fe_2_O_4_ (CuCoF2) and CuFe_2_O_4_ (CuF3) nanoparticles were synthesized using the sonochemical method. The CuCoF1 and CuCoF2 exhibited typical ferromagnetic features, whereas CuF3 displayed superparamagnetic characteristics. The maximum value of EMI total shielding effectiveness (SE_T_) was noticed to be 42.9 dB, 46.2 dB, and 58.8 dB for CuCoF1-rGO-TPU, CuCoF2-rGO-TPU, and CuF3-rGO-TPU nanocomposites, respectively, at a thickness of 1 mm. The highly efficient EMI shielding performance was attributed to the good impedance matching, conductive, dielectric, and magnetic loss. The demonstrated nanocomposites are promising candidates for a lightweight, flexible, and highly efficient EMI shielding material.

## 1. Introduction

Electromagnetic interference, which is generated by the rapid procreation of electronic and communication technology devices, has become a serious concern in everyday life [1,2]. The transmission of electromagnetic waves from potential sources, such as mobile phones, radar systems, and different electronic appliances, are cause for interfering with electronic devices, which influences the lifetime and functionality of such electronic instruments [3]. Therefore, to resolve this problem, the designing of a shielding material endowed with efficient shielding characteristics has become a considerable research interest. Traditional metal-based composites present with many demerits, such as high density, poor corrosion resistance, high processing cost, etc. Moreover, in the case of conventional metals, attenuation of incident electromagnetic (EM) wave occurs by reflection, which has very little contribution towards the reduction of EM pollutions. The material should contain electrical and magnetic dipoles to be an EM wave absorber [4]. In this context, polymer nanocomposites along with magnetic and dielectric nanofillers opened a new pathway due to their lightweight, flexibility, good absorption, low cost, and resistance to corrosion [5]. Epoxy resin, thermoplastic polyurethane (TPU), polyvinylidene fluoride (PVDF), and polydimethylsiloxane are some of the most commonly used polymer matrices [6,7]. Among them, TPU based composites have attained an incredible research interest due to their flexibility, stretchability, superior mechanical properties, and wearable resistance [8]. For example, Valentini et al. used exfoliated graphite-TPU nanocomposite and recorded remarkable shielding efficiency in the microwave region [9]. Similarly, Zahid et al. [10] fabricated nanocomposite based on reduced graphene oxide (rGO) and TPU matrix and reported a high shielding performance of 53 dB. Moreover, Sobha et al. [11] recorded the EMI shielding efficiency of 31.5 dB by utilizing multi-walled carbon nanotube-based TPU composites.

The magnetic properties of spinel ferrite nanoparticles make them an ideal filler for the development of robust electromagnetic shielding polymer nanocomposite material. Moreover, the incorporation of rGO as a second filler along with magnetic spinel ferrites can help in the enhancement of interfacial polarization, high electrical conductivity, and good impedance matching. In our recent work, we demonstrated excellent EMI shielding with MnFe_2_O_4_ and rGO in a polypropylene matrix [12]. Further, the research group of Kumar et al. [13] reported total shielding effectiveness of ≈ 38.2 dB for NiFe_2_O_4_ and rGO nanocomposite in the X-band frequency range. Among all spinel ferrites, there are several studies on CoFe_2_O_4_ along with rGO nanocomposites in a polymer matrix for applications in EMI shielding, which proves CoFe_2_O_4_ as a potential candidate. Dey et al. [14] investigated the EMI shielding efficiency of Co_0.5_Zn_0.4_Cu_0.1_Fe_2_O_4_-GO/paraffin wax hybrid nanocomposite and reported a shielding efficiency of 53.2 dB in the X-band frequency region. Gulzar et al. [8] reported a shielding efficiency of 35 dB in the frequency range from 0.1 to 8 GHz for cobalt ferrites along with coal-fly in the TPU matrix. In addition, Ismail et al. [15] investigated EM shielding and microwave absorption properties of CoFe_2_O_4_ and polyaniline doped with para toluene sulfonic acid and reported a maximum return loss of −28.4 dB at 8.1 GHz.

In this contribution, we utilized hybrid filler systems of Cu_x_Co_1-x_Fe_2_O_4_ (x = 0.33, 0.67, 1) spinel ferrite nanoparticles and rGO inside a TPU matrix for developing nanocomposites with light weight, good flexibility, and highly efficient electromagnetic shielding performance. For the development of thermoplastic polyurethane nanocomposites with reduced graphene oxide and spinel ferrite nanoparticles, we aimed to utilize three samples of spinel ferrite nanoparticles, one with a low content of Cu^2+^, another with a high content of Cu^2+^, and a last one of pure CuFe_2_O_4_ nanoparticles. Therefore, x = 0.33, 0.67, and 1 was selected for Cu_x_Co_1-x_Fe_2_O_4_ spinel ferrite system. To the best of our knowledge, this is the first report on Cu_x_Co_1-x_Fe_2_O_4_ (x = 0.33, 0.67, 1) spinel ferrite nanoparticles along with rGO in the TPU matrix for this purpose.

## 2. Results and Discussion

### 2.1. XRD Study

**Figure 1:**
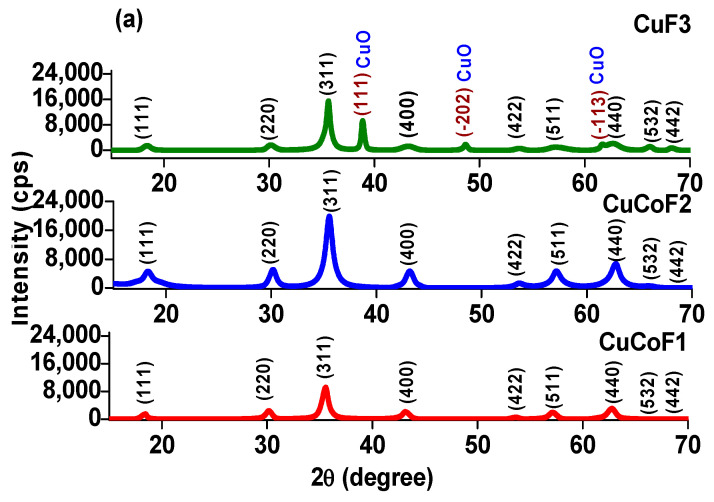

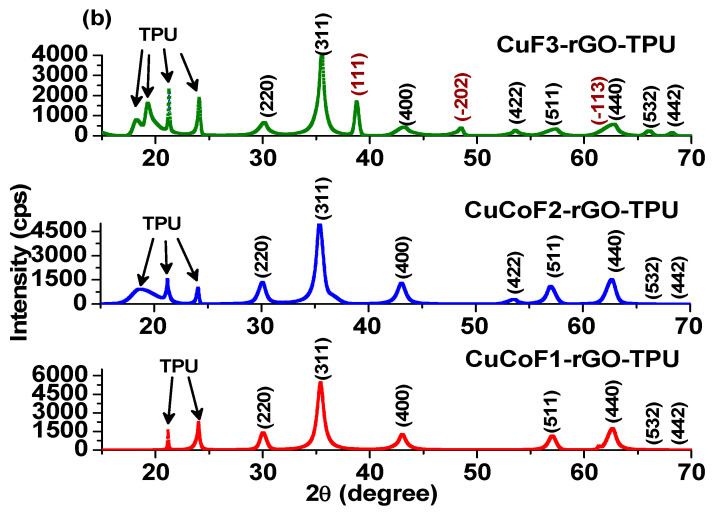
XRD pattern of (**a**) CuCoF1, CuCoF2 and CuF3 nanoparticles (**b**) nanocomposites CuCoF1, CuCoF2 and CuF3 nanoparticles along with rGO in TPU matrix.

Figure 1a depicts the X-ray diffraction (XRD) pattern of CuCoF1, CuCoF2, and CuF3 nanoparticles. The distinctive XRD peaks at 2*θ* = 18.2°, 30.2°, 35.5°, 43.2°, 53.4°, 57°, 62.6°, 66.2°, and 68.3° can be seen, which are assigned to crystalline planes (111), (220), (311), (400), (422), (511), (440), (532) and (442), respectively [16]. The diffraction pattern revealed the monophasic formation of the inverse spinel ferrite crystal structure with space group Fd-$\bar{3}$m in all the samples [17]. However, in the case of the CuF3 sample, some additional peaks of CuO indexed to (111), (−202), and (−113) diffraction planes were also noticed [18]. In the sonochemical synthesis of CuF3 samples, there is an excess concentration of Cu^2+^ in reaction solution, which agglomerates with NaOH under ultrasonic waves and formation of CuO occurs with rising the temperature of the reaction mixture up to 85 °C in sonochemical approach. M.A. Shilpa Amulya et al. [19] also noticed the formation of CuO phases with CuFe_2_O_4_ during the calcination process. Further, the presence of CuO can improve the EMI performance by improving the impedance matching of CuF3-rGO-TPU nanocomposites. Shangyu Gao et al. [20] also noticed the role of the secondary phase on the EMI performance of magnesium alloy. In addition, Lulu Song et al. [21] adjusted the electromagnetic wave absorption characteristics with control of multiple phases.

The crystallite size of the synthesized spinel ferrite nanoparticles was evaluated using Scherrer’s formula:

(1) $$D=\frac{0.94\;\lambda }{\beta Cos\theta }$$

where *D* is the crystallite size (nm), β is the full width of diffraction line at half maxima (in radians), *λ* is the wavelength of the source (Cu-Kα radiation) and θ signifies diffraction angle. The value of crystallite size was calculated for the most prominent peak corresponding to the d_311_ diffraction plane and was found to be 5.51 nm, 4.96 nm, and 3.94 nm for CuCoF1, CuCoF2, and CuF3 nanoparticles, respectively [22,23]. Jnaneshwara et al. [24] also reported a similar trend in crystallite size with the substitution of Cu^2+^ ions in the CoFe_2_O_4_ lattice.

The XRD diffraction pattern for TPU polymer nanocomposites based on CuCoF1, CuCoF2, and CuF3 nanoparticles along with rGO is shown in Figure 1b. As can be seen, the diffraction planes of the face-centered cubic structure of spinel ferrites were present in all the prepared nanocomposite samples [25]. Further, the signature peak for rGO was not present due to its fine dispersion and small size [26]. TPU exhibits broad diffraction peaks ranging from 18° to 24° associated with a mixture of the ordered structure of the hard phase and disordered structure of the amorphous phase [27]. In the case of CuCoF1-rGO-TPU nanocomposite, a distinct peak at 2θ = 21.08° and another peak at 23° remarked the presence of TPU [28]. After the addition of more crystalline CuCoF2 in TPU, the ordering is further improved with appearance of additional peak at 18.7°. In addition, ordering was further improved with addition of CuF3 consisted of CuO phase, and additional peak at 19.2° was noticed. S. Kumar et al. [29] also noticed the appearance of additional peaks with an improvement in the crystallinity of TPU with the addition of MWCNT. The pure TPU exhibited a broad amorphous diffraction peak centered at about 2*θ* = 19.7° of the (110) reflection plane with the interchain spacing of 4.44 Å [30,31].

### 2.2. Raman Study

**Figure 2:**
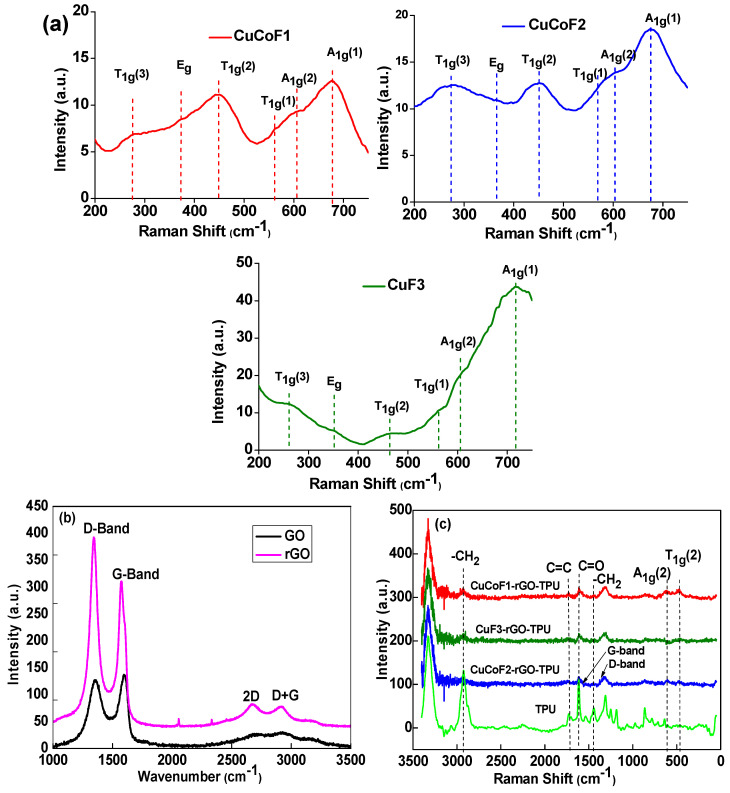
Raman Spectra of (**a**) CuCoF1, CuCoF2 and CuF3 nanoparticles (**b**) GO and rGO (**c**) CuCoF1, CuCoF2, and CuF3 nanoparticles based nanocomposites along with rGO in TPU matrix.

Raman spectroscopy was employed for further investigation of the structural characteristics of prepared nanoparticles and nanocomposites. Figure 2a displays the Raman spectra for CuCoF1, CuCoF2, and CuF3 spinel ferrite nanoparticles. As can be seen, spinel ferrite nanoparticles exhibited Raman bands around 276 cm^−1^, 371 cm^−1^, 463 cm^−1^, 561 cm^−1^, 604 cm^−1^, and 674 cm^−1^ attributed to T_1g_(3), E_g_, T_1g_(2), T_1g_(1), A_1g_(2), and A_1g_(1) Raman modes [32,33]. The lower frequency modes are a consequence of vibration at the tetrahedral site while higher frequency modes reflect vibrations at the octahedral site of spinel ferrite lattice [32,34]. Further, Figure 2b represents the Raman spectra for GO and rGO. Two distinct peaks attributed to the D band at 1353 cm^−1^ and G-band at 1596 cm^−1^ can be noticed for GO. The D vibration band arises from the imperfection edges, also known as the breathing mode of j-point photons of A_1g_ symmetry [35]. However, the peak assigned to the G band is a consequence of the first-order scattering of E_2g_ phonons by sp^2^ carbon at the Brillouin zone center [36]. Similarly, the Raman peaks for D and G bands in Raman spectra were obtained at 1342 cm^−1^ and 1573 cm^−1^ for rGO [37]. Moreover, the intensity ratio (I_D_/I_G_) which is a measure of the extent of the disorder, was noted to be increased after GO was reduced to rGO. The intensity ratio of I_D_/I_G_ was found to be 0.95 and 1.26 for GO and rGO, respectively. In addition, after the reduction of GO, the highest intensity was observed in the case of the D-band [38,39]. Besides the D and G vibration band, other Raman bands at 2674 cm^−1^ and 2911 cm^−1^ assigned to 2D and D+G band were also observed for rGO [40]. The 2D band was related to the inelastic scattering of phonons whereas D+G is the consequence of the summation of both D and G-bands [41].

Figure 2c depicts the Raman spectrum of spinel ferrite nanocomposites with rGO in the TPU matrix. As can be seen, the signature peaks at 2932 cm^−1^, 1533 cm^−1^, 1730 cm^−1^, and 1445 cm^−1^ attributed to stretching vibration of –CH_2_, amide (II), C=O stretching, and bending vibration of –CH_2_ confirmed the existence of polyurethane in nanocomposites [42]. Further, two characteristic peaks at 1342 cm^−1^ and 1573 cm^−1^ attributed to the D and G band of rGO confirmed the presence of rGO in all the composite samples [43].

A slight peak at 606 cm^−1^ assigned to the A_1g_(2) mode of spinel ferrite was noticed [32]. Another Raman band observed at 467 cm^−1^ is assigned with the T_1g_ (2) mode of stretching. The intensity of the peaks is reduced due to the interaction of nanofillers in polyurethane. These modes associated with space group Fd-$\bar{3}$m are the signature of a cubic inverse spinel structure, thus confirming the presence of a spinel ferrite structure inside the nanocomposites [32]. Raman spectroscopy provides confirmation of the presence of spinel ferrite nanoparticles and reduced graphene oxide (rGO) in the thermoplastic polyurethane (TPU) matrix.

### 2.3. Fourier Transform Infra-Red Spectroscopy (FTIR) Analysis

**Figure 3:**
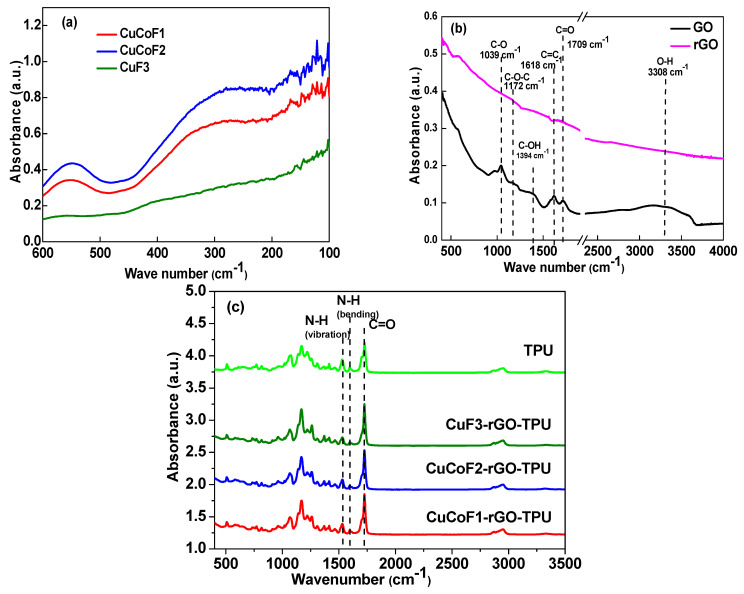
FTIR spectra of (**a**) CuCoF1, CuCoF2 and CuF3 nanoparticles (**b**) GO and rGO (**c**) CuCoF1, CuCoF2 and CuF3 nanoparticles based nanocomposites along with rGO in TPU matrix.

FTIR spectroscopy was employed to investigate the structural characteristics of spinel ferrite nanoparticles, RGO, and its TPU nanocomposites. Figure 3a displays the information on vibrational spectra of CuCoF1, CuCoF2, and CuF3 nanoparticles. One absorption band ʋ_1_ at around 550 cm^−1^ is associated with the intrinsic symmetric vibration due to the metal-oxygen bond at the tetrahedral site and the other absorption band ʋ_2_ around 302–322 cm^−1^ is relevant to octahedral metal stretching [44,45]. The presence of the two major absorption bands ʋ_1_ and ʋ_2_ in FTIR spectra, as shown in Figure 3a, confirmed the cubic spinel structure of all the nanoparticles samples [46,47]. The obtained results are in good agreement with the reported literature [48].

The presence of oxygen-containing functional groups was confirmed by FTIR spectra of GO, represented in Figure 3b. As can be observed, the absorption bands at 1709 cm^−1^ and 1618 cm^−1^ represent the carbonyl stretching (C=O) and skeletal stretching (C=C) of the alkene group [49,50]. Moreover, the high intensity of prominent peaks in GO divulges the presence of a large amount of oxygen-containing functional groups. Furthermore, another absorption band centered around 1000–1100 cm^−1^ assigned to the C-O epoxide group was also noticed [51]. A broad absorption band between 2500 and 3500 cm^−1^ was attributed to the carboxyl (–COOH) groups [52]. It can be observed in Figure 3b that, after the reduction of GO, there was a significant decrease in the intensity of absorption spectra of alkoxy and hydroxyl groups [53]. Indeed, all attributed peaks possess weaker intensity as compared to the intensity of the FTIR spectrum of GO, which proves the successful reduction of GO.

Figure 3c depicts the FTIR spectra of CuCoF1, CuCoF2, and CuF3 nanocomposites along with rGO in the TPU matrix. As can be seen in the graph, a slight absorption peak at 3329 cm^−1^ for all the samples is attributed to the stretching vibration of -NH in the urethane group [42]. Moreover, the characteristic bands of TPU regardless of synthesis route were also noticed at 1725 cm^−1^ and 1527 cm^−1^ ascribed to C=O stretching and N-H bending vibration in polyurethane, thus confirming the existence of TPU in nanocomposites [54]. In consolidation with Raman and FTIR spectra analysis, the existence of spinel ferrite nanoparticles and rGO in TPU was confirmed.

### 2.4. TEM and HRTEM Analysis of Nanoparticles

**Figure 4:**
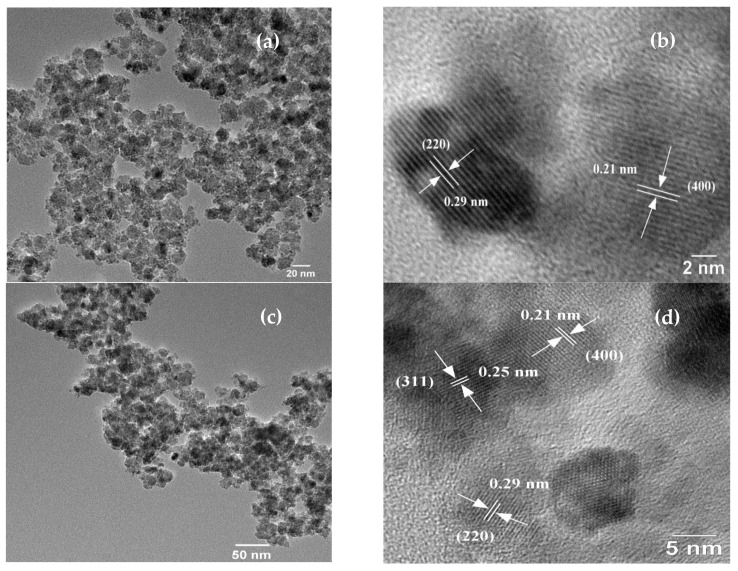

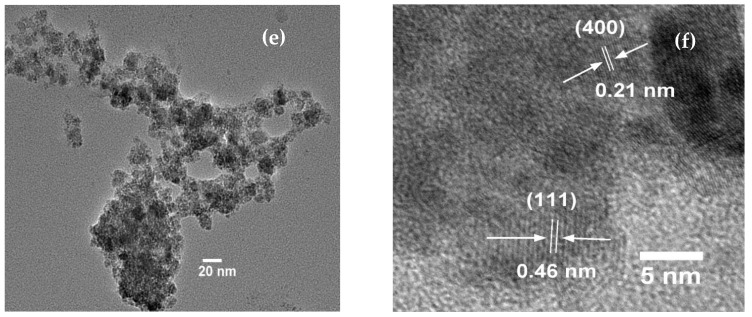
(**a**) TEM and (**b**) HRTEM of CuCoF1; (**c**) TEM, and (**d**) HRTEM of CuCoF2; (**e**) TEM and (**f**) HRTEM image of CuF3.

Figure 4 depicts the TEM and HRTEM images of CuCoF1, CuCoF2, and CuF3 nanoparticles. The spherical nanoparticles of 3–5 nm with slight agglomeration can be noticed from Figure 4a for the CuCoF1 sample. The HRTEM image of the CuCoF1 sample as shown in Figure 4b, displays lattice of (220) planes (d spacing 0.29 nm) and (400) planes (d spacing 0.21 nm) of spinel ferrite [55]. Further, the TEM image (Figure 4c) of the CuCoF2 sample shows spherical particles of 2–4 nm with moderate agglomeration. The lattice of (311) planes (d spacing 0.25 nm), (220) planes (d spacing 0.29 nm), and (400) planes (d spacing 0.21 nm) of spinel ferrite can be observed in the HRTEM image (Figure 4d) for the CuCoF2 sample. Furthermore, spherical nanoparticles of 2–3 nm can be observed in Figure 4e for the CuF3 sample. The HRTEM image of CuF3 as displayed in Figure 4f, reveals the lattice of (400) planes (d spacing 0.21 nm) and (111) planes (d spacing 0.46 nm) of spinel ferrite.

### 2.5. FE-SEM and EDX Study of Nanocomposites

**Figure 5:**
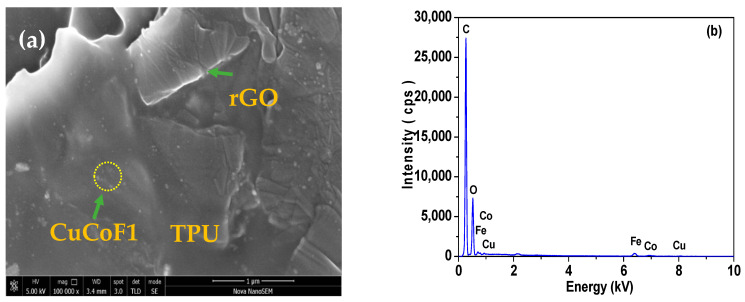

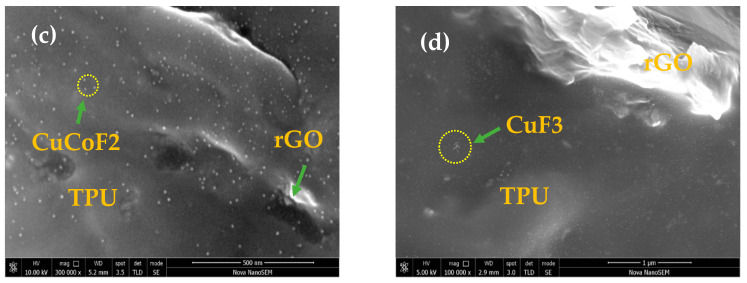
(**a**) FE-SEM image of CuCoF1-rGO-TPU, (**b**) EDX pattern of CuCoF1-rGO-TPU, (**c**) FE-SEM image of CuCoF2-rGO-TPU, and (**d**) FE-SEM image of CuF3-rGO-TPU.

To investigate the nanofillers in the TPU matrix, FE-SEM images were studied. Figure 5a depicts the FE-SEM image of the cryo-fractured surface of CuCoF1-rGO-TPU nanocomposite which depicts the dispersion of CuCoF1 and rGO nanofillers in the TPU matrix. Figure 5b shows the EDX spectrum of CuCoF1-rGO-TPU nanocomposite, which confirm the presence of Cu, Co, Fe, C, and O. Further, the good dispersion of spinel ferrite nanoparticles and rGO in TPU matrix of other prepared nanocomposites CuCoF2-rGO-TPU, and CuF3-rGO-TPU can be seen in FE-SEM image, as shown in Figure 5c,d, respectively.

### 2.6. Magnetic Property

**Figure 6:**
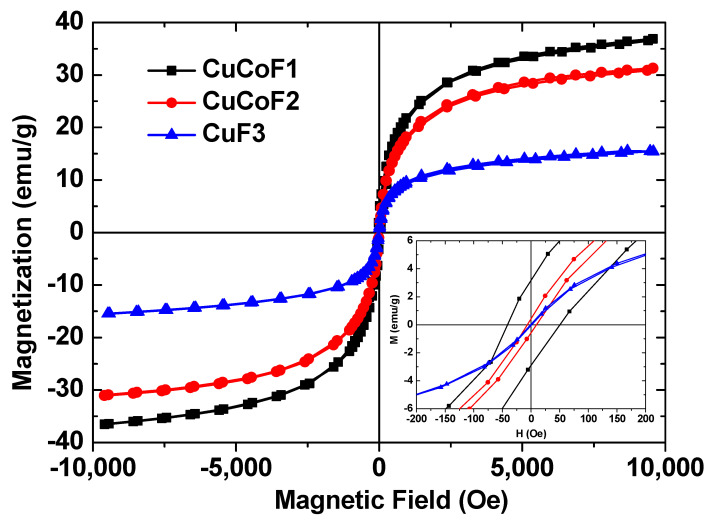
Magnetic hysteresis curves of prepared CuCoF1, CuCoF2, and CuF3 spinel ferrite nanoparticles.

Figure 6 depicts the magnetic hysteresis curves of prepared CuCoF1, CuCoF2, and CuF3 spinel ferrite nanoparticles. The magnetic hysteresis curves of CuCoF1, and CuCoF2 show typical ferromagnetic features. The value of saturation magnetization (M_s_) of the CuCoF1, CuCoF2, and CuF3 samples are 37.2 emu/g, 31.5 emu/g, and 15.6 emu/g, respectively. The decrease in the value of M_s_ with a decrease of grain size is associated with an increase of the surface spin canting and dead magnetic layer [56]. The decrease in saturation magnetization (M_s_) with an increase of Cu^2+^ content can also be explained by the probable replacement of Co^2+^ by Cu^2+^ at the tetrahedral sites of the spinel ferrite lattice systems [57]. The observed variation in M_s_ can be explained by the help of Neel’s two sub-lattice magnetization model [58]. According to this model, magnetization is obtained with the help of $M\left(\mu_{B}\right)=M_{B}-M_{A}$, where *M_A_* and *M_B_* are the net magnetic moment of tetrahedral (A) and octahedral (B) sites, respectively. The decrease in the saturation magnetization can be attributed to the lower magnetic moment of Cu^2+^ (1$\mu_{B}$) than Co^2+^ (3$\mu_{B}$). Thus, the magnetic moment in the B-sublattice was sequentially decreased with the increase in copper substitution, which results in lower magnetic moment of copper substituted spinel ferrite nanoparticles [59]. Similar results were noticed by other researchers [57,58,59]. The remanent (M_r_) magnetization values were 3.2 emu/g, 0.48 emu/g, and 0 emu/g for CuCoF1, CuCoF2, and CuF3 sample, respectively. The coercivity (H_c_) value was 50.4 Oe, 9.5 Oe, and 0 Oe for CuCoF1, CuCoF2, and CuF3 samples, respectively. In nanosized particles, a single magnetic domain with no residual magnetism are known as superparamagnetic characteristics [60]. Below a critical size of a ferromagnetic material, the anisotropy energy is lower than the thermal energy, which leads to superparamagnetic characteristics [61]. The magnetic hysteresis curve of CuF3 display an S-shape with no coercivity and remanence, which points to the superparamagnetic characteristic, and can be beneficial for utilization as high-performance electromagnetic interference shielding material at high frequency [62].

### 2.7. Electromagnetic Interference Shielding

Electromagnetic interference shielding is a mechanism of reflection and absorption of electromagnetic radiation by a material that prevents the penetration of harmful electromagnetic waves. It is defined as the ratio of incident power (P_i_) to the outgoing power (P_t_) of electromagnetic waves and is represented in the decibel (dB) unit. The mechanisms which contribute to total shielding effectiveness, SE_T_, are shielding due to reflection SE_R_, absorption SE_A_, and multiple internal reflections SE_M_. The total shielding effectiveness SE_T_ can be mathematically written as follows [63]:

$${\mathrm{SE}}_{T}=-10\log(P_{t}/P_{i})={\mathrm{SE}}_{R}+{\;\mathrm{SE}}_{A}+{\;\mathrm{SE}}_{M}$$

The reflection of EM wave is due to the impedance mismatch between the air and the absorber (shielding material) by mobile charge carriers such as electrons and holes whereas in the case of multiple reflections, the scattering effect is responsible due to inhomogeneity and large interfacial area inside the matrix [64]. Absorption is associated with the dissipation of EM waves in form of heat energy through shielding material. Moreover, several other factors such as the thickness of the shielding material, ohmic loss, polarization loss, and magnetic loss have their role. Ohmic losses in a shielding material are related to the dissipation of energy due to charge hopping, tunneling, and conducting mechanisms. Polarization losses originate due to the dissipation of energy required for overcoming the state of reorientation of dipoles in every half-cycle of EM radiations caused by functional groups, interfaces, and defects in material [64]. The main magnetic losses are associated with the eddy current loss, natural resonance, hysteresis loss, and exchange resonance [65]. Although according to Schelkunoff’s theory, if the shielding exceeds 10 dB and the shielding material has thickness higher than the material skin depth (δ), then the role of multiple reflections (SE_M_) can be disregarded in practical EMI applications and only SE_A_ and SE_R_ are taken into consideration for the total shielding effectiveness SE_T_ [64]. Therefore, the higher is the value of SE_T_, the lower the energy transmission through a shielding material will be.

**Figure 7:**
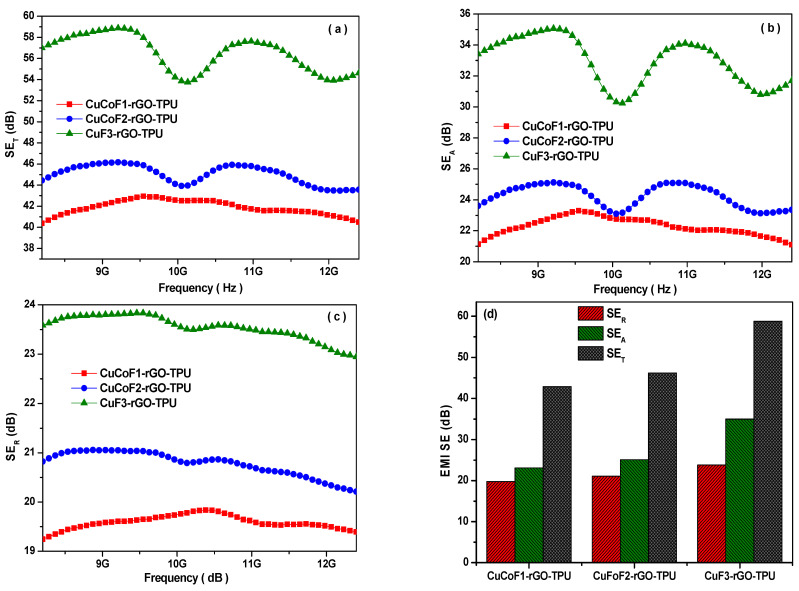
Electromagnetic interference shielding effectiveness (**a**) due to SE_T_ (**b**) SE_R_ (**c**) SE_A_ (**d**) comparison chart of the value of SE_R_, SE_A_, and SE_T_ for CuCoF1, CuCoF2, and CuF3 nanoparticles based nanocomposites along with rGO in TPU matrix.

In the present study, we investigated the electromagnetic interference shielding effectiveness of CuCoF1-rGO-TPU, CuCoF2-rGO-TPU, and CuF3-rGO-TPU nanocomposites in the X-band frequency (8.2–12.4 GHz), as depicted in Figure 7a. As can be seen, the maximum value of SE_T_ was found to be 42.9 dB, 46.2 dB, and 58.8 dB for CuCoF1-rGO-TPU, CuCoF2-rGO-TPU, and CuF3-rGO-TPU nanocomposites, respectively. Next, Figure 7b elaborates effective shielding performance more in terms of another electromagnetic parameter which is absorption, SE_A_. As observed, the maximum value of SE_A_ was 23.1 dB, 25.1 dB, and 35.0 dB for developed CuCoF1-rGO-TPU, CuCoF2-rGO-TPU, and CuF3-rGO-TPU nanocomposites, respectively. For further assessment, the value of SE_R_ was also evaluated. Figure 7c represents the values of SE_R_, which were found to be 19.8 dB, 21.1 dB, and 23.8 dB for CuCoF1-rGO-TPU, CuCoF2-rGO-TPU, and CuF3-rGO-TPU nanocomposites, respectively. Notably, the high value of SE_A_ in these nanocomposites indicates that the absorption of EM waves is the dominant mechanism. The plot in Figure 7d displays an evaluation and comparison of electromagnetic shielding parameters. As can be seen, the highest values of SE_T_, SE_A_, and SE_R_ for the CuF3-rGO-TPU nanocomposite signify that this composition possesses good electromagnetic shielding properties. In addition, EMI shielding efficiency (%), which signifies the capability of shielding material to block EM waves in terms of percentage, can be evaluated by the following relation with the EMI shielding effectiveness (dB) [66]:

$$\mathrm{Shielding}\;\mathrm{Efficiency}\;\left(\%\right)=100-\left(\frac{1}{10^{\frac{\mathrm{SE}}{10}}}\right)\times 100$$

The EMI shielding efficiency (%) was 99.9948%, 99.9976%, and 99.9998% for CuCoF1-rGO-TPU, CuCoF2-rGO-TPU, and CuF3-rGO-TPU nanocomposites, respectively. It signifies that CuF3-rGO-TPU nanocomposite has a blockage of 99.9998% of the incident EM waves with only 0.0002% transmission. In addition, the shielding ability, as well as the lightweight property of material, can be expressed by specific shielding effectiveness, SSE (=EMISE/density) and the absolute shielding effectiveness, SSE/t (=SSE/thickness) [67]. The evaluated value of SSE was 90.232 dB cm^3^ g^−1^ and SSE/t was 902.320 dB cm^2^ g^−1^ for CuF3-rGO-TPU nanocomposite.

A research group, Ali et al. [68] investigated microwave absorption characteristics of Polyaniline (PANI)/NiZn ferrite nanocomposites by varying ferrite percentage and reported a maximum reflection loss of −39.56 dB with 2.5 mm sample thickness. Similarly, another research group Kumar et al. [13] studied the electromagnetic shielding behavior of NiFe_2_O_4_/rGO possessing a thickness of 2.0 mm and observed SE_T_ of 38.2 dB in the X-band frequency range. Further, a maximum value of SE_T_ of 53 dB by fabrication of rGO-TPU nanocomposite has been reported by Zahid et al. [10]. Furthermore, Gunasekaran et al. [69] observed an optimum reflection loss RL of −31.89 dB with rGO/zirconium substituted cobalt ferrite (Co_0.5_Zr_0.5_ Fe_2_O_4_) nanocomposites having 5 mm sample thickness. Moreover, Gahlout et al. [70] studied electromagnetic shielding response for polypyrrole-MWCNT/polyurethane composites and reported a SE_T_ of 48 dB with a sample thickness of 3 mm in the X-band frequency range. Furthermore, Sulaiman et al. [71] reported excellent absorption characteristics of −24.86 dB for Co_0.5_Zn_0.5_ Fe_2_O_4_/PANI-PTSA nanocomposites (weight ratio 1:1) with an optimal matching thickness of 3 mm. Total shielding effectiveness, SE_T_ of 35 dB for Polystyrene/PANI/Nickel spinel ferrite composite, is noticed in a broad frequency range of 0.1 to 20 GHz by Shakir et al. [72]. Moreover, Li et al. [73] reported total shielding effectiveness SE_T_ of 48.4 dB with nitrogen-doped reduced graphene oxide/CoFe_2_O_4_ hybrid nanocomposites in X and Ku bands. The recent advances in the electromagnetic shielding performance of some nanocomposites are displayed in Table 1.

### 2.8. Electromagnetic Parameters

**Figure 8:**
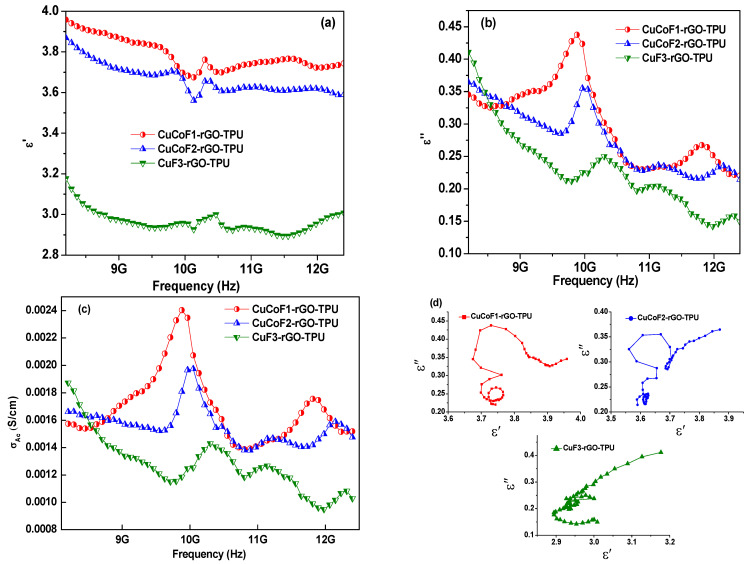
(**a**) the real part (ε′) (**b**) the imaginary part (ε″) of permittivity (**c**) ac conductivity (σ_ac_) versus frequency, and (**d**) Cole-cole plots for CuCoF1, CuCoF2 and CuF3 nanoparticles based nanocomposites along with rGO in TPU matrix.

For further evaluation and better comparison with different compositions of polymer nanocomposites, we investigated intrinsic parameters such as complex permittivity and complex permeability. In both complex parameters, the real term attributes to energy storage, while the imaginary term attributes to loss or energy dissipation within the material contributed from conduction, resonance, and relaxation mechanisms [74]. The graph in Figure 8a demonstrates the variation of the real part of permittivity, ε′ with a change in the frequency from 8.2 to 12.4 GHz for CuCoF1-rGO-TPU, CuCoF2-rGO-TPU, and CuF3-rGO-TPU nanocomposites. As can be seen, the value of ε′ was found to vary from 3.67 to 3.95, 3.56 to 3.85, and 2.89 to 3.16 for CuCoF1-rGO-TPU, CuCoF2-rGO-TPU, and CuF3-rGO-TPU nanocomposites, respectively. Various kinds of polarization such as interfacial polarization, dipolar polarization, electronic polarization, and space charge polarization contribute to the value of ε′ [75,76]. For studying the dielectric loss of the material, the imaginary part of permittivity (ε″) is significant. The graph in Figure 8b depicts the variation of imaginary permittivity (ε″) with change in frequency for Cu_x_Co_1-x_Fe_2_O_4_ (x = 0.33, 0.67, and 1.00)-rGO TPU nanocomposites. As can be seen, the value of ε″ was found to be altering from 0.23 to 0.44, 0.21 to 0.36, and 0.14 to 0.41 for CuCoF1-rGO-TPU, CuCoF2-rGO-TPU, and CuF3-rGO-TPU nanocomposites, respectively. The imaginary part of permittivity provides information about energy dissipation. In the ε″ curves, the resonance peaks can be attributed to the leakage conductance and lags in polarization [77,78].

It is worth noting that ε″ and electrical conductivity are dependent on each other with the following expression [79]:

$$\sigma_{\mathrm{ac}}=\varepsilon_{o}\varepsilon^{\prime \prime }2\pi f$$

where σ_ac_ denotes electrical conductivity, ε_o_ stands for the absolute permittivity of vacuum, f represents the frequency of electromagnetic waves. The ac conductivity (σ_ac_) with change in frequency of CuCoF1-rGO-TPU, CuCoF2-rGO-TPU, and CuF3-rGO-TPU nanocomposites at room temperature is displayed in Figure 8c. As can be seen, the value σ_ac_ is in the range 1.40 × 10^−3^–2.41 × 10^−3^ S/cm, 1.36 × 10^−3^–1.96 × 10^−3^ S/cm, and 9.44 × 10^−4^–1.87 × 10^−3^ S/cm for CuCoF1-rGO-TPU, CuCoF2-rGO-TPU, and CuF3-rGO-TPU nanocomposites, respectively. The Debye theory of relaxation is employed for an explanation of the mechanism involved in the electromagnetic shielding material. According to the Debye theory, the value of ε′ and ε″ can be expressed as follows [80]:

$$\varepsilon^{\prime }=\varepsilon_{\infty }+\frac{\varepsilon_{s}-\varepsilon_{\infty }}{1+{\left(\omega \tau \right)}^{2}}$$

$$\varepsilon^{\prime \prime }=\frac{\varepsilon_{s}-\varepsilon_{\infty }}{1+{\left(\omega \tau \right)}^{2}}\omega \tau +\frac{\sigma }{{\omega \varepsilon }_{o}}$$

where ε_∞_ represents the relative dielectric permittivity at an infinite frequency, ε_s_ signifies the static dielectric permittivity, ω denotes angular frequency, and τ is the polarization relaxation time, respectively. On neglecting the role of σ to ε″ and removal of ωτ, the equation between ε′ and ε″ can be deduced as follows:

$${\left(\varepsilon^{\prime }-\frac{\varepsilon_{s}+\varepsilon_{\infty }}{2}\right)}^{2}+{\left(\varepsilon^{\prime \prime }\right)}^{2}={\left(\frac{\varepsilon_{s}-\varepsilon_{\infty }}{2}\right)}^{2}$$

According to the above expression, the Cole–Cole plot which is ε″ versus ε′ should be a semicircle. Each Debye relaxation process possesses a semi-circle that can be upgraded through the interface, subsequently improving the tendency of EM absorption [81]. Figure 8d displays the Cole–Cole plots for CuCoF1-rGO-TPU, CuCoF2-rGO-TPU, and CuF3-rGO-TPU nanocomposites. As can be seen, at least three Cole–Cole semicircles are present which marks the role of multiple relaxation mechanisms involved in synthesized nanocomposites. Moreover, the semi-circles were found to be distorted, which suggests that apart from Debye relaxation, there could be involvement of other mechanisms in the nanocomposites, such as the presence of interfaces leading to interfacial polarization or the Maxwell Wagner effect in the developed samples [82].

**Figure 9:**
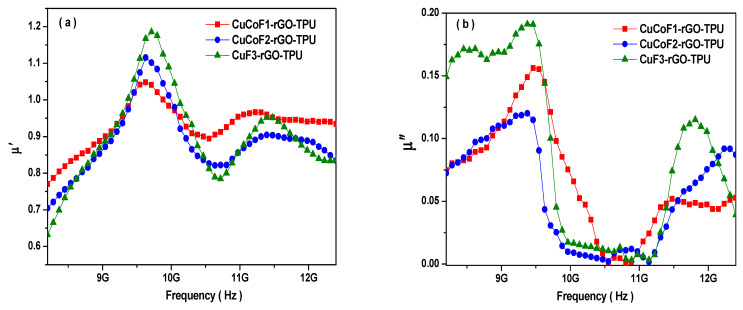

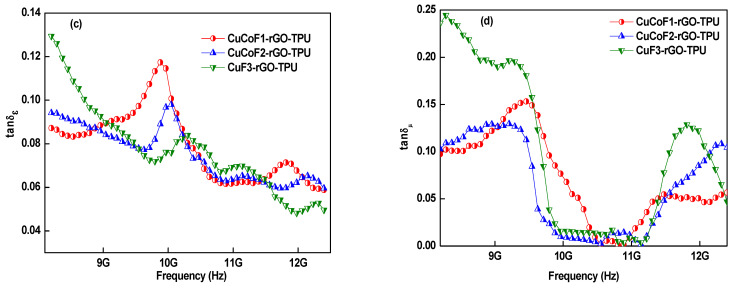
Variation of (**a**) the real part (µ′) of permeability, (**b**) the imaginary part (µ″) of permeability, (**c**) dielectric loss, tanδ_ε_ (**d**) magnetic loss, tanδ_µ_ with change in frequency for CuCoF1, CuCoF2, and CuF3 nanoparticles based nanocomposites along with rGO in TPU matrix.

Further, for the sake of better evaluation of the EMI shielding mechanism, the real part of permeability (µ′), which signifies the storage capacity of magnetic energy was also studied. Figure 9a displays the plot of µ′ with a change of frequency from 8.2 to 12.4 GHz for Cu_x_Co_1-x_Fe_2_O_4_ (x = 0.33, 0.67, and 1.00)-rGO-TPU nanocomposites. As can be seen, the value of µ′ is varying within the range of 0.77 to 1.04, 0.71 to 1.11, and 0.64 to 1.18 for CuCoF1-rGO-TPU, CuCoF2-rGO-TPU, and CuF3-rGO-TPU nanocomposites, respectively. The imaginary part of permeability (µ″) represents the energy loss of the magnetic field. Figure 9b displays the imaginary part (µ″) of permeability with change in the frequency of Cu_x_Co_1-x_Fe_2_O_4_ (x = 0.33, 0.67, and 1.00)-rGO-TPU nanocomposites. The value of µ″ is found to be varied between 0.00 to 0.15, 0.00 to 0.12, and 0.00 to 0.19 for CuCoF1-rGO-TPU, CuCoF2-rGO-TPU, and CuF3-rGO-TPU nanocomposites, respectively. Three peaks were observed for the value of µ″ in the frequency region of 8.2 GHz to 12.4 GHz for all samples, which correspond to strong natural resonance [81,83].

Dielectric loss plays a vital role in the attenuation of EM waves. To compare dielectric loss capabilities of prepared nanocomposites, it was calculated using the following expression:

$${\tan\delta }_{\varepsilon }=\varepsilon^{\prime \prime }/\varepsilon^{\prime }$$

Figure 9c displays the dielectric loss (tanδε) of Cu_x_Co_1-x_Fe_2_O_4_ (x = 0.33, 0.67, and 1.00)-rGO-TPU nanocomposites. As can be seen, the value of tanδ_ε_ is in the range of 0.06–0.12, 0.06 to 0.10, and 0.05 to 0.13 for CuCoF1-rGO-TPU, CuCoF2-rGO-TPU, and CuF3-rGO-TPU nanocomposites, respectively. It is worth noting that values of ε″ and tanδ_ε_ follow a similar trend with a change in frequency for all the nanocomposites. The residual groups and defects in rGO contribute to dielectric loss in nanocomposites. In addition, as a result of polarization and associated relaxation, dielectric loss is developed in nanocomposites [84,85]. For further analysis, the magnetic loss was calculated for nanocomposites, which is given by the following expression

$${\tan\delta }_{µ}={µ}^{\prime \prime }/{µ}^{\prime }$$

Figure 9d below displays the variation of magnetic loss tanδ_µ_ with change in frequency. As can be seen, the value of tanδ_µ_ varies from 0.00 to 0.15, 0.00 to 0.13, and 0.00 to 0.24 for CuCoF1-rGO-TPU, CuCoF2-rGO-TPU, and CuF3-rGO-TPU nanocomposites, respectively. The high value of tanδ_µ_ compared with tanδ_ε_ reveals that magnetic loss is dominant in endowing for electromagnetic characteristics. It is well known that the magnetic loss, tanδ_µ_ arises due to eddy current, natural resonance, and anisotropic energy present inside the polymer nanocomposite [86]. The magnetic loss induced by eddy current loss can be evaluated using the following equation [87]:

$$C_{o}={µ}^{\prime \prime }{\left({µ}^{\prime }\right)}^{-2}f^{-1}$$

**Figure 10:**
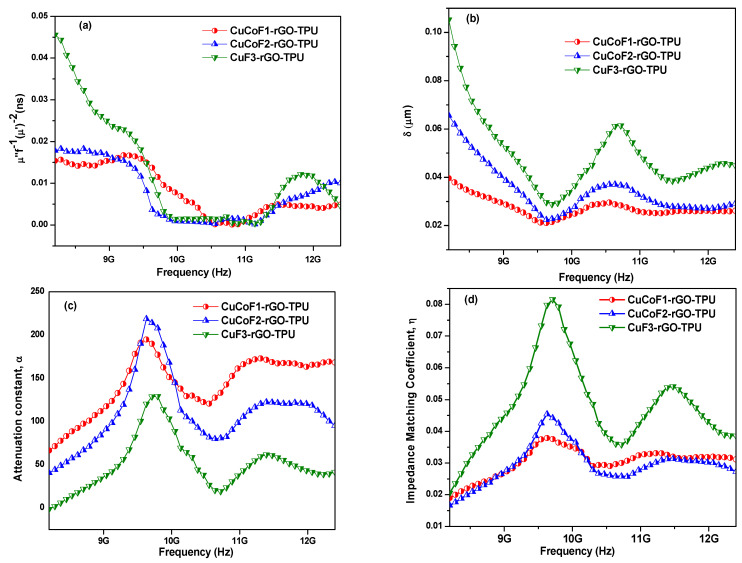
Variation of (**a**) eddy current loss, C_o_ (**b**) skin depth, δ (**c**) attenuation constant, α and (**d**) impedance matching, Z with change in frequency for CuCoF1, CuCoF2, and CuF3 nanoparticles based nanocomposites along with rGO in TPU matrix.

In general, if the value of C_o_ is not changing with the change in the frequency, then it is obvious that magnetic loss is induced by the eddy current. Figure 10a depicts the plot of C_o_ with change in frequency over X-band region for CuCoF1-rGO-TPU, CuCoF2-rGO-TPU, and CuF3-rGO-TPU nanocomposites. As can be seen, the value of C_o_ with the variation of frequency remains nearly constant with change in frequency from 9.7 GHz to 11.2 GHz for CuCoF2-rGO-TPU and CuF3-rGO-TPU nanocomposites. It shows that eddy currents are a contributor to the magnetic loss for CuCoF2-rGO-TPU and CuF3-rGO-TPU nanocomposites. However, in the case of CuCoF1-rGO-TPU nanocomposite, the value of C_o_ was found constant in the frequency range 8.3–8.8 GHz, 10.4–10.9 GHz, and 11.4–12.3 GHz, which signifies that eddy current is contributing to the magnetic loss in this frequency range [88].

The EM wave enters only near the surface of the shielding material at higher frequencies and the strength of the EM field suffers exponential decay with the thickness. The skin depth (δ) of shielding material refers to the certain distance up to which the strength of the electric field suffers attenuation and drops to 1/e of its original incident EM wave [89]. Theoretically, it can be represented as follows [90]:

$$\delta =\frac{1}{\sqrt{{\pi \mu }_{r}\sigma f}}$$

where μ_r_ refers to the magnetic permeability of the material, σ is electrical conductivity and f is the frequency. The given plot in Figure 10b represents the variation of skin depth (δ) as a function of frequency for CuCoF1-rGO-TPU, CuCoF2-rGO-TPU, and CuF3-rGO-TPU nanocomposites. The value of skin depth was found to be fluctuating from 0.02 µm to 0.04 µm, 0.022 µm to 0.065 µm, and 0.03 µm to 0.11 µm for CuCoF1-rGO-TPU, CuCoF2-rGO-TPU, and CuF3-rGO-TPU nanocomposites, respectively.

To understand the underlying EMI shielding mechanism in the nanocomposites, attenuation constant (α) and impedance matching conditions were studied. The attenuation constant (α) was determined for calculating the degree of energy attenuated when an EM wave was incident on the surface of the shielding material [91]. The attenuation constant (α) can be expressed by the following equation [92]:

$$\alpha =\frac{\sqrt{2}\pi f}{c}\sqrt{\left({µ}^{\prime \prime }\varepsilon^{\prime \prime }-{µ}^{\prime }\varepsilon^{\prime }\right)+\sqrt{{\left({µ}^{\prime \prime }\varepsilon^{\prime \prime }-{µ}^{\prime }\varepsilon^{\prime }\right)}^{2}+{\left(\varepsilon^{\prime }{µ}^{\prime \prime }+\varepsilon^{\prime \prime }{µ}^{\prime }\right)}^{2}}}$$

where c stands for speed for light in vacuum. Figure 10c represents the attenuation constant (α) of the developed for CuCoF1-rGO-TPU, CuCoF2-rGO-TPU, and CuF3-rGO-TPU nanocomposites with variation in frequency. It was observed that CuCoF1-rGO-TPU possesses maximum attenuation constant (α) value as compared with other developed nanocomposites. However, only attaining a high value of attenuation constant doesn’t mark for good absorption characteristics. A good and balanced impedance match should be achieved between the permittivity and permeability of the shielding material [93]. Keeping this in mind, the impedance matching (Z) was also evaluated for different nanocomposites using the following relation [94]:

$$Z=\sqrt{{µ}_{r}/\varepsilon_{r}}=\sqrt{\sqrt{\left({{µ}^{\prime }}^{\;2}+{{µ}^{\prime \prime }}^{\;2}\right)}/\sqrt{\left({\varepsilon^{\prime }}^{\;2}+{\varepsilon^{\prime \prime }}^{\;2}\right)}}$$

**Figure 11:**
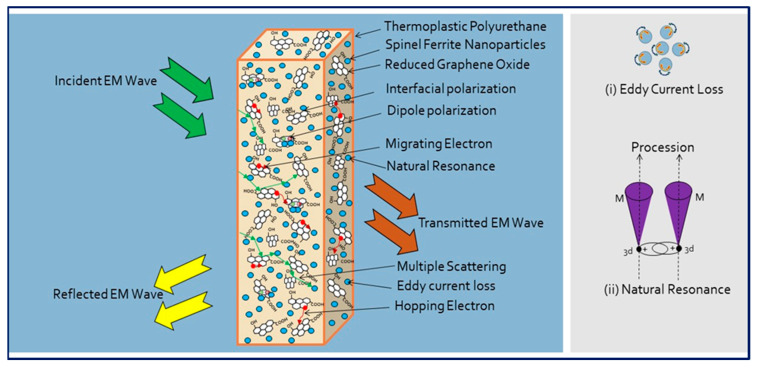
Schematic illustration of EMI shielding mechanism based on developed Cu_x_Co_1-x_Fe_2_O_4_ (x = 0.33, 0.67, and 1.00)-rGO-TPU nanocomposites.

The impedance matching (Z) with change in frequency is displayed in Figure 10d. Notably, the highest value of impedance matching (Z) for CuF3-rGO-TPU nanocomposite as compared with other developed nanocomposites was noticed. Owing to the balanced impedance matching and moderate attenuation capacity of CuF3-rGO-TPU nanocomposite, it comes up with good shielding performance [95]. Referring to the above discussion, a possible EMI shielding mechanism based on developed Cu_x_Co_1-x_Fe_2_O_4_ (x = 0.33, 0.67, and 1.00)-rGO-TPU nanocomposites has been proposed and illustrated in Figure 11. When EM waves interact with developed nanocomposite, some EM waves are immediately reflected due to the presence of abundant free electrons on the surface [96]. The remaining EM waves move through the shielding material due to a good impedance matching condition. In this process, the EM waves are attenuated because of the dielectric loss, conduction loss, magnetic loss, and multiple scattering/reflection. In this developed nanocomposite, under the interaction of EM waves, the dielectric loss is mainly associated with interfacial polarization and dipole polarization. The interfacial polarization arises from interfaces between Cu_x_Co_1-x_Fe_2_O_4_ spinel ferrite nanoparticles and rGO. The dipole polarization derives from defects and residual functional groups of rGO [97]. Magnetic Cu_x_Co_1-x_Fe_2_O_4_ spinel ferrite nanoparticles induce magnetic loss characteristics and contribute to the absorption component of EMI shielding. Magnetic loss is mainly associated with eddy current loss and natural resonance [98]. The high conductivity of rGO sheets in favor of intrinsic or hopping conduction causes the enhancement of conduction loss. The synergistic effect between Cu_x_Co_1-x_Fe_2_O_4_ spinel ferrite nanoparticles and rGO with an efficient complementarity between the complex permeability and permittivity provides a good impedance matching condition [99]. Hence, the synergy of multiple loss mechanisms associated with good impedance matching provides CuF3-rGO-TPU nanocomposite with high EMI shielding performance.

## 3. Materials and Methods

### 3.1. Chemicals

Cobalt nitrate Co(NO_3_)·6H_2_O, copper nitrate Cu(NO_3_)·6H_2_O, and iron nitrate Fe(NO_3_)_3_·9H_2_O were purchased from Alfa Aesar GmbH & Co. KG (Karlsruhe, Germany). Sodium nitrate (NaNO_3_) was obtained from Lach-Ner, Czech Republic. Potassium permanganate (KMnO_4_) powder and graphite flakes were sourced from Sigma-Aldrich, Munich, Germany. Vitamin C (Livsane) was a product of Dr. Kleine Pharma GmbH, Bielefeld, Germany.

### 3.2. Synthesis of Cu_x_Co_1-x_Fe_2_O_4_ (x = 0.33. 0.67, 1) Spinel Ferrite Nanoparticles

Cu_x_Co_1-x_Fe_2_O_4_ (x = 0.33, 0.67, 1) spinel ferrite nanoparticles with different compositions, such as Cu_0.33_Co_0.67_Fe_2_O_4_, Cu_0.67_Co_0.33_Fe_2_O_4_, and CuFe_2_O_4_, labelled as CuCoF1, CuCoF2, and CuF3, were synthesized using the sonochemical method. For Cu_x_Co_1-x_Fe_2_O_4_ (x = 0.33. 0.67, 1) spinel ferrite nanoparticle formation, appropriate stoichiometric amounts of cobalt nitrate, copper nitrate, and iron nitrate were taken and mixed with 60 mL of deionized water in a 100 mL beaker and stirred for 15 min at room temperature. A solution of NaOH was prepared and added to the above mixture slowly accompanying stirring for a further 2–3 min. The solution turned into a thick precipitate with the addition of a base solution. Further, it was exposed to ultrasonic irradiation (ultrasonic homogenizer UZ SONOPULS HD 2070) (frequency: 20 kHz and power: 70 W) for 60 min. Afterward, the precipitate was cooled down, washed with deionized water, and further centrifuged at 7000 rpm for 15 min. This process was repeated several times to remove any remaining impurities. The acquired product was further dried at 60 °C in an oven for 24 h.

### 3.3. Synthesis of Graphene Oxide

Graphene oxide (GO) was synthesized following the modified Hummer’s method [100] using graphite flakes as a raw material. For this purpose, 3 g of graphite and 1.5 g of NaNO_3_ were mixed with 75 mL of H_2_SO_4_ (98%) in a 1000 mL flask in an ice bath (0 °C) and was stirred for 15 min. Next, 9 g of KMnO_4_ was added slowly and carefully during the vigorous magnetic stirring for 30 min. After that, the prepared mixture was again subject to magnetic stirring for 30 min. Further, the reaction temperature was fixed to room temperature and the mixture was stirred for an additional 48 h. Next, 138 mL of deionized water was added to the mixture, which was followed by stirring for 10 min with heating at 100 °C. Then, another 420 mL of warm deionized water and 30 mL of H_2_O_2_ were added to the mixture. Afterward, the synthesized yellow suspension was washed with an aqueous solution of H_2_SO_4_ (6 wt%) and H_2_O_2_ (1 wt%). Next, the prepared suspension was subjected to washing with deionized water until the pH turned neutral, and then the mixture was three times centrifuged at 6000 rpm for 10 min. The precipitate was then dried in the oven at 50 °C for 10–12 h to obtain graphite oxide powder. To utilize GO as a filler in TPU nanocomposite with spinel ferrite nanoparticles, the enhancement of the electrical conductivity of GO is required. The electrical conductivity of GO can be improved by doping or reduction of oxygen functional groups [101]. In the present work, the improvement of the electrical conductivity of GO via reduction of the oxygen functional group was achieved by a chemical approach with vitamin C as reducing agent.

### 3.4. Synthesis of Reduced Graphene Oxide

Reduced graphene oxide (rGO) was obtained by chemical reduction of synthesized graphene oxide (GO). Firstly, 3 g of graphene oxide (GO) was dissolved in 200 mL of deionized water in a 500 mL flask and the solution was stirred for 15 min. Next, 10 g of vitamin C was added, and the solution was stirred for 3 h to a fixed temperature of 100 °C. The mixture was cooled down and then washed with deionized water and ethanol. After that, it was centrifuged at 8000 rpm for 20 min. The product was then dried in a vacuum oven at 60 °C for 14 h.

### 3.5. Preparation of Nanocomposites

**Figure 12:**
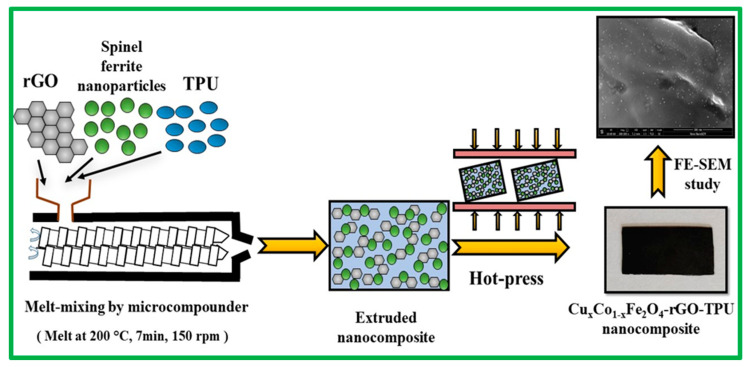
Schematic representation of the preparation of Cu_x_Co_1-x_Fe_2_O_4_-rGO-TPU nanocomposite.

**Figure 13:**
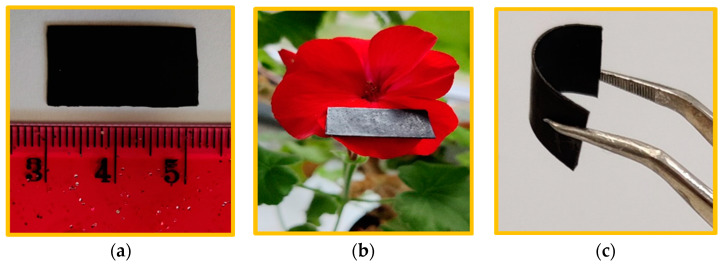
(**a**–**c**) Digital photograph for the demonstration of dimension, lightweight, and flexibility of prepared CuF3 and rGO based TPU nanocomposite.

For the preparation of thermoplastic polyurethane (TPU) based nanocomposites of 20 wt% nanofiller (in which spinel ferrites nanoparticles and rGO was in 9:1 wt% ratio) were mixed with TPU (Elastollan^®^ C80A10) in A microcompounder (Xplore Instruments B.V., Sittard, The Netherlands) with a capacity of 5 cm^3^. TPU and fillers were dried at 90 °C for 12 h in a vacuum oven before mixing. The samples were melt-mixed in a microcompounder at 200 °C for 7 min at 150 rpm. The force was varied from 2405 Newton to 1680 Newton during preparation of nanocomposites. Further, the nanocomposite characteristics, such as electrical, mechanical, thermal, and optical, etc., have dependence on interfacial physical and chemical interactions [102]. The strong interfacial interactions between nanofillers (spinel ferrite and rGO) and polymer matrix, may provide high nanocomposites properties [103]. Three nanocomposite systems, namely CuCoF1-rGO-TPU, CuCoF2-rGO-TPU, and CuF3-rGO-TPU, were prepared. The rectangular-shaped sheets of 22.9 mm × 10.2 mm × 1.0 mm were developed by compression molding. Further, the schematic representation of the preparation of Cu_x_Co_1-x_Fe_2_O_4_-rGO-TPU nanocomposite is shown in Figure 12. Furthermore, a digital photograph for the demonstration of the dimension, lightweight, and flexibility of a prepared nanocomposite is shown in Figure 13.

## 4. Conclusions

In this work, lightweight and flexible TPU polymer matrix-based nanocomposites embedded with Cu_x_Co_1-x_Fe_2_O_4_ (x = 0.33, 0.67, and 1.00) spinel ferrite nanoparticles and rGO as nanofillers were fabricated by a melt-mixing approach using a microcompounder, which demonstrated remarkable shielding effectiveness values. Spinel ferrite Cu_0.33_Co_0.67_Fe_2_O_4_ (CuCoF1), Cu_0.67_Co_0.33_Fe_2_O_4_ (CuCoF2), and CuFe_2_O_4_ (CuF3) nanoparticles were developed by a facile sonochemical synthesis method. The developed nanocomposites with a thickness of 1 mm exhibited maximum total shielding effectiveness (SE_T_) of 42.9 dB, 46.2 dB, and 58.8 dB for CuCoF1-rGO-TPU, CuCoF2-rGO-TPU, and CuF3-rGO-TPU nanocomposites, respectively. It was found that 99.9998% of electromagnetic waves can be shielded in the investigated frequency range, which indicates that the CuF3-rGO-TPU nanocomposite can be considered an effective electromagnetic shielding material. With abundant interfacial polarization, dipole relaxation, better impedance matching condition, and eddy current effect, the natural resonance of nanocomposites plays a key role in this outstanding EMI shielding performance. It is believed that the current investigation could be beneficial in the design and development of lightweight and flexible shielding materials with outstanding EMI shielding performance.

## Figures and Tables

**Table 1:** EMI shielding characteristics of nanocomposites reported in some previous literatures.

Shielding Material	Frequency Band	Sample Thickness	SE_T_	Ref.
NiFe_2_O_4_/rGO	8.2–12.4 GHz	2 mm	38.2 dB	[13]
rGO-TPU nanocomposite	0.1–20 GHz	250 µm	53 dB	[10]
TPU-PCNT composites	8.2−12.4 GHz	3.0 mm	48 dB	[70]
Polystyrene/PANI/Nickel spinel ferrite composite	0.1–20 GHz	0.25 mm	35 dB	[72]
Nitrogen-doped reduced graphene oxide/CoFe_2_O_4_ hybrid nanocomposites	8–18 GHz	1.9 mm	48.4 dB	[73]
CuFe_2_O_4_-rGO-TPU	8.2–12.4 GHz	1 mm	58.8 dB	This work

## Data Availability

The data will be available from the corresponding author following reasonable request.
